# Novel Wearable System to Recognize Sign Language in Real Time

**DOI:** 10.3390/s24144613

**Published:** 2024-07-16

**Authors:** İlhan Umut, Ümit Can Kumdereli

**Affiliations:** 1 Department of Electronics and Automation, Corlu Vocational School, Tekirdag Namik Kemal University, Tekirdag 59850, Türkiye; 2 Department of Computer Engineering, Faculty of Engineering, Trakya University, Edirne 22030, Türkiye; ucankumdereli@trakya.edu.tr

**Keywords:** artificial intelligence, computer software, human–computer interaction, inertial measurement unit, sign language recognition, surface electromyography

## Abstract

The aim of this study is to develop a practical software solution for real-time recognition of sign language words using two arms. This will facilitate communication between hearing-impaired individuals and those who can hear. We are aware of several sign language recognition systems developed using different technologies, including cameras, armbands, and gloves. However, the system we propose in this study stands out for its practicality, utilizing surface electromyography (muscle activity) and inertial measurement unit (motion dynamics) data from both arms. We address the drawbacks of other methods, such as high costs, low accuracy due to ambient light and obstacles, and complex hardware requirements, which have limited their practical application. Our software can run on different operating systems using digital signal processing and machine learning methods specific to this study. For the test, we created a dataset of 80 words based on their frequency of use in daily life and performed a thorough feature extraction process. We tested the recognition performance using various classifiers and parameters and compared the results. The random forest algorithm emerged as the most successful, achieving a remarkable 99.875% accuracy, while the naïve Bayes algorithm had the lowest success rate with 87.625% accuracy. The new system promises to significantly improve communication for people with hearing disabilities and ensures seamless integration into daily life without compromising user comfort or lifestyle quality.

## 1. Introduction

The World Health Organization reports that around 466 million individuals have hearing impairments, with this number expected to rise to 700 million by 2050 [1]. Sign language uses hands, facial movements, and body posture to express thoughts, feelings, and information instead of verbal communication. There are several types of sign languages with alphabets and signs used worldwide. TSL, like other sign languages, has a different word order and grammar than Turkish. Although hearing-impaired individuals use sign language to communicate with each other, they may face difficulties when interacting with others. As a result, the prevalence of psychological problems among them is high [2]. Writing and reading are standard methods of communication between hearing people and people who are deaf or hard of hearing. Writing is the most effective method of communicating with a person with hearing impariment, mainly when accuracy is crucial. However, in some situations, writing may not be feasible. Speechreading is another method of communication, but many spoken Turkish sounds do not appear on the lips. Therefore, a real-time sign language recognition (SLR) system is necessary to translate signs into sound or text.

Although the focus of our paper is on SLR, most of the work in this area consists of hand gesture recognition. Hand gestures are used to express only letters and numbers. However, communicating in this way can be slow and difficult. It is much faster and easier to sign words using hand, arm, and facial expressions instead of individual letters [3]. Studies have been conducted on the subject and solutions have been developed. The majority of these studies have focused on specific sign languages, particularly American Sign Language (ASL) and Chinese Sign Languages (CSLs) [3]. Various technologies have been employed, including Microsoft Kinect [4,5], Leap Motion [6,7,8,9,10,11], data gloves [12,13], cameras [14,15,16,17,18,19,20,21,22,23,24,25], surface electromyography (sEMG), and inertial measurement unit (IMU) [26,27,28,29,30,31,32,33,34,35,36]. Although some systems have achieved high levels of accuracy, none are likely to be suitable for everyday real-life situations. Refer to Table 1 [37] for a comparison of current technology-based systems.

Motion data in image-oriented techniques is acquired when using a camera, while solutions using data gloves acquire position, direction, and velocity information of movement through sensors. Although most developers prefer image-based techniques for gesture recognition, these techniques have several disadvantages. For instance, gestures are highly dependent on perspective, which can cause different gestures to appear the same to a poor-quality passive camera. Furthermore, image-based methods necessitate consistent lighting and fixed camera placements while requiring high processing power, memory, and energy. Although Leap Motion, a different visual-based method, has advantages over cameras, it remains theoretical since it must be in a fixed position.

In solutions where sensors are used, the user wears gloves or armbands containing various sensors to detect movements instead of relying on images. Fels et al. [38] are pioneering teams in glove-based SLR methods. They achieved good classification success (94%) using artificial neural networks to classify 203 ASL signals. However, wearing gloves is uncomfortable under normal conditions, and technical obstacles limit the use of this method in laboratory environments. When comparing armbands and gloves, it is essential to note that armbands can help reduce sweating and provide a more aesthetically pleasing appearance. They also require fewer sensors and can communicate wirelessly. Furthermore, armband systems that use sEMG and IMU technology are popular due to their affordability and portability. IMU sensors are specifically used to capture arm movements. sEMG sensors, on the other hand, are suitable for acquiring movement information of hands and fingers [39]. For instance, although some signs in TSL share similarities in hand and arm movements, the movements of the fingers differ. This makes it challenging to differentiate between IMU data alone and these movements. However, with the aid of sEMG data, finger movements can also be distinguished, enabling the classification of signs. In a study on CSL recognition, accelerometers and sEMG sensors were employed together. The study used a hierarchical decision tree as the classification algorithm and achieved 95.3% accuracy [40]. The study most similar to ours was conducted by Wu et al. [41]. They developed a system that utilized a wrist-worn IMU and four sEMG sensors to recognize 80 different ASL signs. The study achieved an average accuracy of 96.16% using a support vector machine classifier. However, their hardware was a prototype, and they did not have software like ours, which can obtain and classify data simultaneously. Therefore, their study remained theoretical.

This study aims to develop software for real-time SLR using two Myo armbands. The system offers critical contributions regarding practicality, affordability, and mobility. Unlike many current technologies that are limited to controlled environments, this system is designed to be used in everyday real-life situations. The system utilizes Myo armbands, which are compact, light, and less costly than other technologies, enhancing mobility and reducing user inconvenience associated with more cumbersome devices. Our research did not find a comparable system appropriate for daily use.

## 2. Materials and Methods

### 2.1. System Hardware

**Figure 1:**
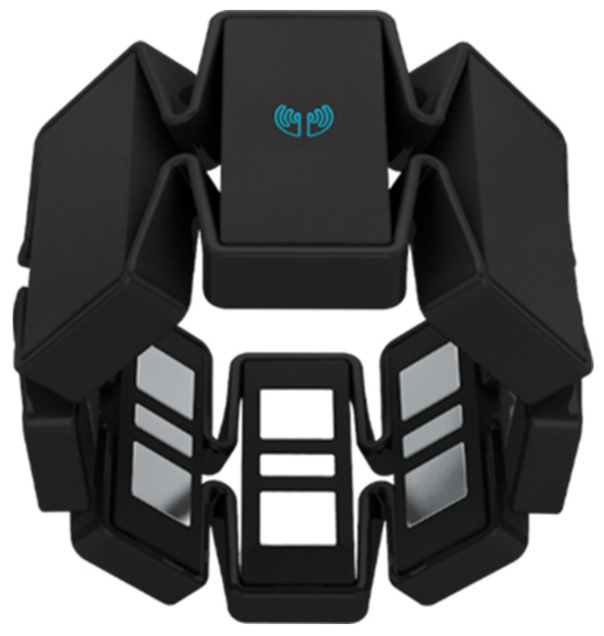
Myo armband.

Data from the arm was collected using two Myo armbands from Thalmic Labs, each consisting of an IMU and sEMG sensors [42]. The device is depicted in Figure 1.

Myo is a commercial motion controller that contains an ARM Cortex-M4 120 MHZ microprocessor, eight dry sEMGs with a sampling rate of 200 Hz, and a nine-axis IMU with a sampling rate of 50 Hz. The IMU provides ten different types of data. The accelerometers in the device measure acceleration in terms of the gravitational constant (G), while the gyroscope measures rotation in terms of rad/s. The device includes magnetometers to obtain orientation and positioning measurements, which are used to determine the movement of the arm. Additionally, the device has eight dry sEMG sensors that allow for the detection of finger movements.

The Myo armband was the ideal choice for the study outlined in this manuscript due to its exceptional features that make it particularly suited for real-time SLR. The Myo armband was selected for its integrated sensors, including IMU and sEMG sensors. The IMU sensors capture arm movements, while the sEMG sensors detect muscle activity related to hand and finger movements. This combination provides comprehensive data necessary for accurate gesture recognition. The Myo armbands are wireless and highly portable, allowing for practical, everyday use outside laboratory environments. Additionally, the armbands enable real-time processing of the captured data. The Myo armband processes data in real time, making it ideal for translating sign language into text or speech with minimal delays. The sensors have a high resolution, ensuring precise muscle activity and motion dynamics detection. This study’s results demonstrate that the random forest algorithm achieved an accuracy rate of 99.875%. The Myo armband design is user-friendly and comfortable to wear, which is crucial for ensuring user acceptance and continuous use. It can be effortlessly put on and taken off and does not necessitate the user to handle complex setups. Compared to other motion capture devices such as data gloves and camera-based systems, Myo armbands are a cost-effective option. This technology is highly accessible and practical for large-scale deployment thanks to its enhanced accessibility. The Myo armband boasts a robust application programming interface (API) that empowers the creation of tailored applications, such as the SLR system mentioned in this manuscript. This support allows for developers to personalize applications to suit the unique demands of their projects. The Myo armband is an excellent choice for developing an SLR system that is effective and practical for everyday use. It addresses many of the limitations found in previous technologies.

### 2.2. Developed Software

**Figure 2:**
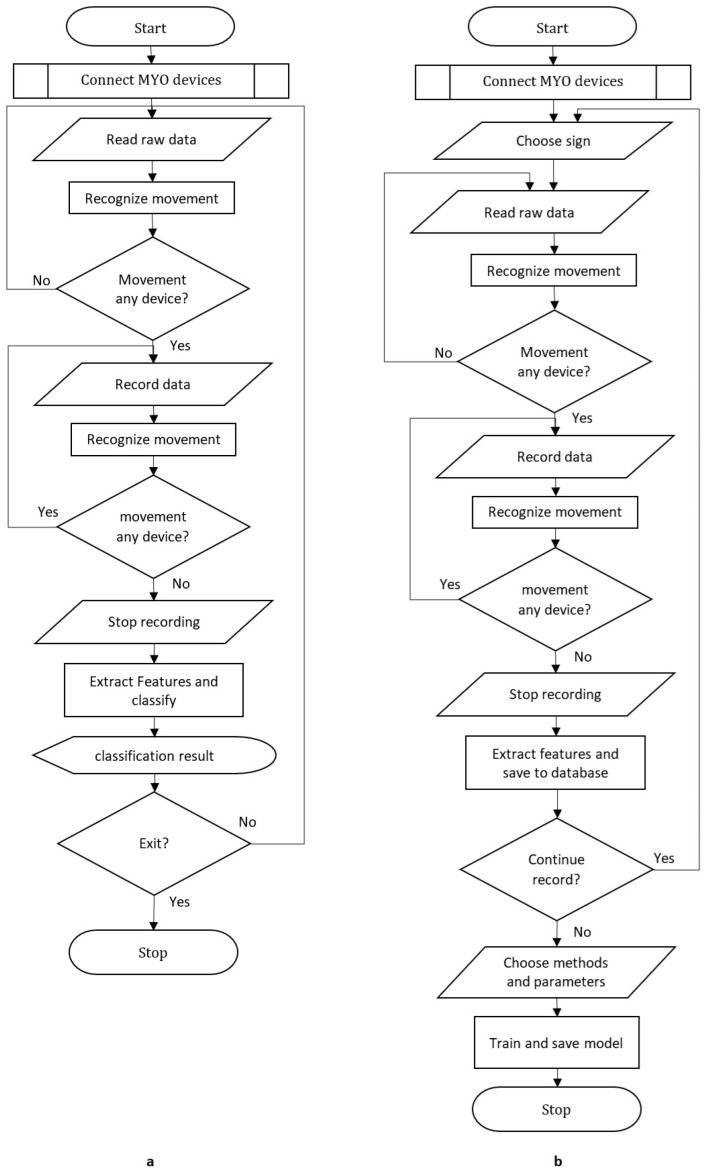
Flow chart of the software: (**a**) test and (**b**) train.

The application was developed using the Delphi programming language and Embarcadero RAD Studio 11 Integrated Development Environment. Two versions were created, for Windows and Android platforms, with the possibility of creating additional versions for Linux and IOS platforms using the same code. SQLite was used as the database. Figure 2 presents a flowchart outlining the sequential steps of the proposed methodology for recognizing sign language using the Myo armband. A detailed description of Figure 2 using pseudocode is shown below. On the left is the test and on the right is the description of the training algorithm.
**START** **CONNECT MYO devices** *// Check Bluetooth and location services then connects the MYO devices to the system.* **: BEGIN** **DO**  **Read raw data** *// Read raw data form connected devices (IMU and sEMG).*  **Recognize movement** *// Is there a change above the threshold value in any or both values   taken from the two devices?* **WHILE movement recognized** *// If motion is detected, proceed to the recording cycle* **DO**  **Read raw data**  **Record data** *// Save raw data to memory*  **Recognize movement** **WHILE no movement is recognized** *// If motion is not detected, stop recording and proceed to the classify* **Stop recording** **Extract features and classify** *// Classify with the selected classification algorithm by extracting Time domain, Frequency domain, and Entropy-based features.* **Show result** *// Show classification result. Play with voice via TTS* **IF not exit the app, THEN GO TO BEGIN** *// go to BEGIN if the app is not closed* **STOP****START** **CONNECT MYO devices** **:BEGIN** **Choose sign** *// Select the sign to use in classification training* **DO**  **Read raw data**  **Recognize movement** **WHILE movement recognized** **DO**  **Read raw data**  **Record data**  **Recognize movement** **WHILE not movement recognized** *// If motion is not detected, stop recording and proceed to the train* **Stop recording** **Extract features and save to the database // Extract Time domain, Frequency domain, and entropy-based** *features and save them to the database with the label of the selected sign.* **IF continue record THEN GO TO BEGIN** *// go to BEGIN if record section is not closed.* **Choose methods and parameters.** **Train and save model** *//Perform classification training with the selected algorithm and parameters. Then, save the model as a file* **STOP**

**Figure 3:**
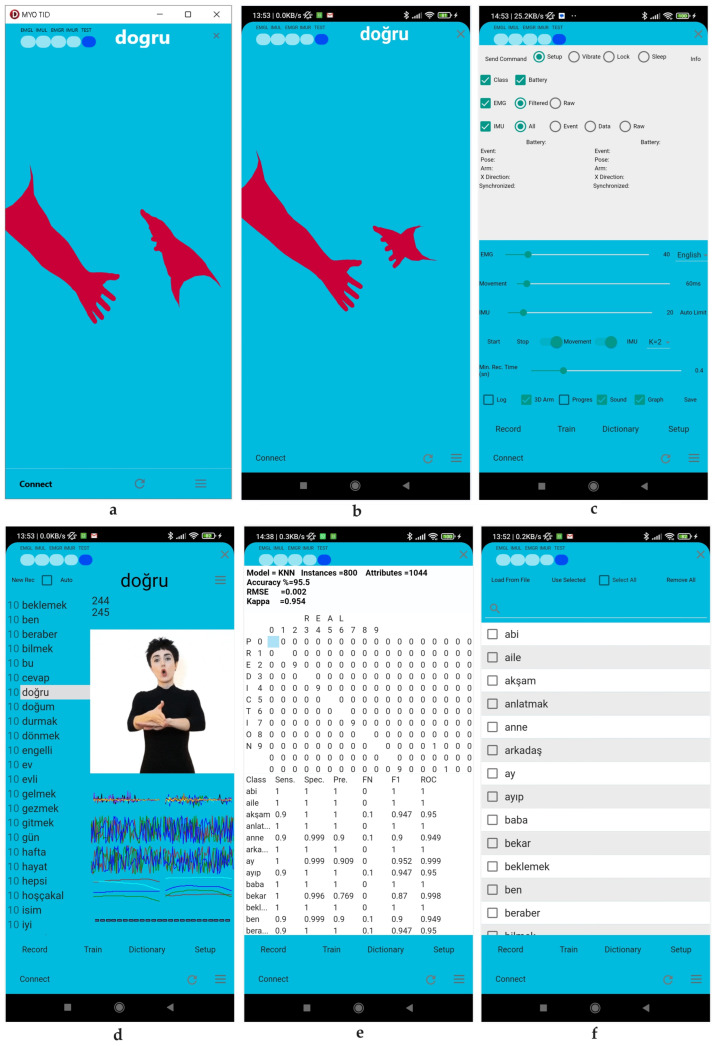
An example of the graphical user interfaces: (**a**) Windows main user interface; (**b**) Android main user interface; (**c**) settings user interface; (**d**) records user interface; (**e**) train user interface; (**f**) dictionary user interface.

The program has five different user interfaces. All interfaces are given in Figure 3.

#### 2.2.1. Main User Interface

Upon running the program for the first time, the user interface depicted in (Windows Figure 3a and Android Figure 3b) will appear. The interface is designed with simplicity in mind. Pressing the connect button triggers the program to check for location and Bluetooth permissions. If permission is not granted, the program will request it from the user. If permission is granted but Bluetooth connection or location access is turned off, the program will turn them on. Finally, the program will establish a connection to both Myo armbands using the Bluetooth Low Energy (BLE) protocol. After establishing the connection, the label and oval-shaped markers on the left of the screen appear if the EMG and IMU services are successfully subscribed. These markers turn blue when there is a change above the threshold value in the data. The blue oval indicator under the TEST label indicates that the program can make predictions from instantaneous movements. The guessed word is displayed in written form in the upper middle of the screen. The color transition in the three-dimensional arms can be used to observe changes in the EMG data of each arm. The battery status of each armband is displayed as a number ranging from 0 (empty) to 100 (fully charged) in the upper left and right corners of the interface. To return the three-dimensional arms to their reference position, click the refresh icon in the lower right corner. Access the settings interface by clicking on the three-line icon located in the lower right corner.

#### 2.2.2. Settings User Interface

The interface (Figure 3c) allows for access and modification of all device and software settings. It is possible to adjust the device’s data, vibration, lock, sleep, and wake functions.

*Data Segmentation Settings:*

Precise data segmentation settings can be adjusted individually. Each EMG and IMU datum is stored in a global variable for segmentation. Motion is detected by calculating the difference between the current and previous measurements of 200 Hz (EMG) and 50 Hz (IMU) data. The user can adjust the limit values in the settings interface to suit their needs.

*Movement Track Bar:* The pause between two signals can be adjusted between 20 ms and 1 s (1000 ms). If you wish to speak quickly, select the minimum value of 20 ms. In the example interface, this value is set to 60 ms. If the limit value specified in the EMG or IMU is not exceeded for 60 ms (12 measurements, each taking 5 ms since the device operates at 200 Hz), the sign is considered finished. Following the end of the sign, the data properties are calculated and sent to the artificial intelligence algorithm for classification.

*EMG Trackbar:* The maximum value of the EMG Trackbar is 256, corresponding to the range of EMG data from the device (−127 to 128). In the example interface, this value is set to 40. Therefore, any absolute change of 40 in the values of the eight EMG sensors indicates motion.

*IMU Trackbar:* The IMU orientation data determines the device’s position in the *x*, *y*, and *z* planes and detects motion. The IMU Trackbar maximum value is set to 360, as this is the maximum value of the angles being measured. In the provided interface, the value is set to 20. If the absolute value of the mean of the changes in the Euler angle labels (*α*, *β*, and *γ*) exceeds 20 degrees, it indicates movement. The Euler angles are obtained from the IMU sensor using the quaternion method. To convert the given Euler angle labels to quadrilateral labels, use the following conversion. The values of roll, pitch, and yaw angles from the Euler angle notation are represented by *α*, *β*, and *γ*, respectively. The assumed rotation order is from pitch to roll from deflection. The corresponding quarter q is defined as follows:

(1) $$q=\left[\begin{array}{c} q_{w}\\ q_{x}\\ q_{y}\\ q_{z} \end{array}\right]=\left[\begin{array}{c} \cos\frac{\alpha }{2}\cos\frac{\beta }{2}\cos\frac{\gamma }{2}+\sin\frac{\alpha }{2}\sin\frac{\beta }{2}\sin\frac{\gamma }{2}\\ \sin\frac{\alpha }{2}\cos\frac{\beta }{2}\cos\frac{\gamma }{2}-\cos\frac{\alpha }{2}\sin\frac{\beta }{2}\sin\frac{\gamma }{2}\\ \cos\frac{\alpha }{2}\sin\frac{\beta }{2}\cos\frac{\gamma }{2}+\sin\frac{\alpha }{2}\cos\frac{\beta }{2}\sin\frac{\gamma }{2}\\ \cos\frac{\alpha }{2}\cos\frac{\beta }{2}\sin\frac{\gamma }{2}-\sin\frac{\alpha }{2}\sin\frac{\beta }{2}\cos\frac{\gamma }{2} \end{array}\right]$$

*Min. Rec. Time Track Bar:* The minimum duration of the movement is set with the minimum record time, which is 0.4 s in the example interface. A certain quantity of data is required to calculate certain features. An option has been added to prevent the software from giving errors.

*Interface Features and Controls*

Additionally, detailed information can be displayed or hidden using checkboxes for Log, 3D Arm, Progress, Sound, and Graph for simplicity and performance improvement.

The Log Checkbox allows for instant viewing of all data and software/device changes.

The 3D Arm Checkbox displays arm movements on the screen in real time. The feature can be hidden to reduce performance and battery consumption.

The Progress Checkbox displays recording and training times as a progress bar.

The signs are read aloud using the operating system’s text-to-speech feature if the Sound Checkbox is selected. This software feature enables the user’s phone to convert sign language movements into sound.

The Graph Checkbox allows for visualization of device data in chart form.

*MYO Armband Classification Features*

**Figure 4:**
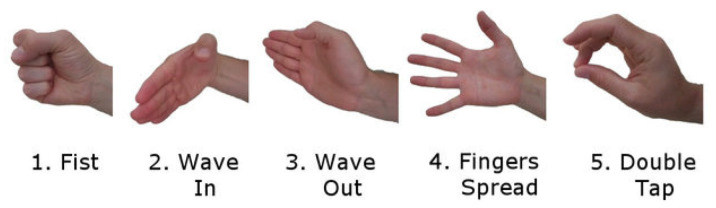
The five gestures recognized by the Myo armband [43].

Additionally, the MYO Armband’s classification features can control the application (e.g., putting the device to sleep, waking it up, and starting and stopping). Figure 4 shows that the device classifies five hand movements as hardware and sends them over the BLE service. The application can also utilize these features to generate ten different commands from two different devices. For instance, when the user makes a ‘Fist’ movement with their right arm, the 3D arms move to the reference position. To display the logs, click on the icon of three lines in a row in the lower right corner of the main graphical interface. The logs will appear when the ‘Wave In’ movement is made with the right arm and disappear when the same movement is made again.

When the ‘Fingers Spread’ movement is made with the right arm, the BLE characteristics of the devices are subscribed, meaning that the data begins to be received. In other words, the program starts. When the movement is repeated, the program stops. This feature enables the user to modify the Movement Track Bar, including adjustments to the speech rate or other application settings, more efficiently. A multitude of commands can be executed via the wristband without the necessity of manual input from the user, even when the phone is stored within a pocket.

#### 2.2.3. Records User Interface

This interface was designed to collect data for training artificial intelligence. Figure 3d displays a list box on the left showing words and the number of records associated with each word. Select the word to create a new record and click the ‘New Rec’ button. Recording begins when the motion starts and continues until it ends. When the recording is complete, the word count increases by one. If the Auto Checkbox is selected, the list box will automatically switch to the next word. When the movement starts, the data for the next word are saved. If it reaches the last word in the list, it will move on to the first word and continue until the Stop Rec button is clicked. This interface allows for a graphical display of data from the device during recording. Additionally, suppose a gif file with the same name as the word exists. In that case, the movement video will be displayed if a gif file with the same name as the word exists. The list box at the top displays the record numbers of the selected words in the database. By selecting the record number, record that record’s data are graphically displayed, allowing for the identification of any incorrect records. To remove unwanted records, double-click (double Tab on the phone) on the record number and select ‘yes’ in the warning message. The interface displays the graph of record number 244 for the word ‘correct’.

#### 2.2.4. Train User Interface

Training involves using the data obtained after extracting features from the IMU and EMG raw data. The algorithm and parameters selected in this interface are used to train existing data. The training is then tested using the 10-fold cross-validation method. The performance values of the end-of-training algorithm are displayed in Figure 3e. The sample interface uses the K-nearest neighbor algorithm with a K = 3 parameter.

#### 2.2.5. Dictionary User Interface

This interface allows for adding and removing words or sentences from the database (refer to Figure 3f). Additionally, words can be uploaded from a txt file for convenience. However, please note that loading from the file will result in deleting all existing records. If accepted, a new dictionary will be added. As the application is personalized, it can create a unique experience by allowing for users to choose their own words, create custom records, and train it using artificial intelligence.

### 2.3. Data Collecting

**Figure 5:**
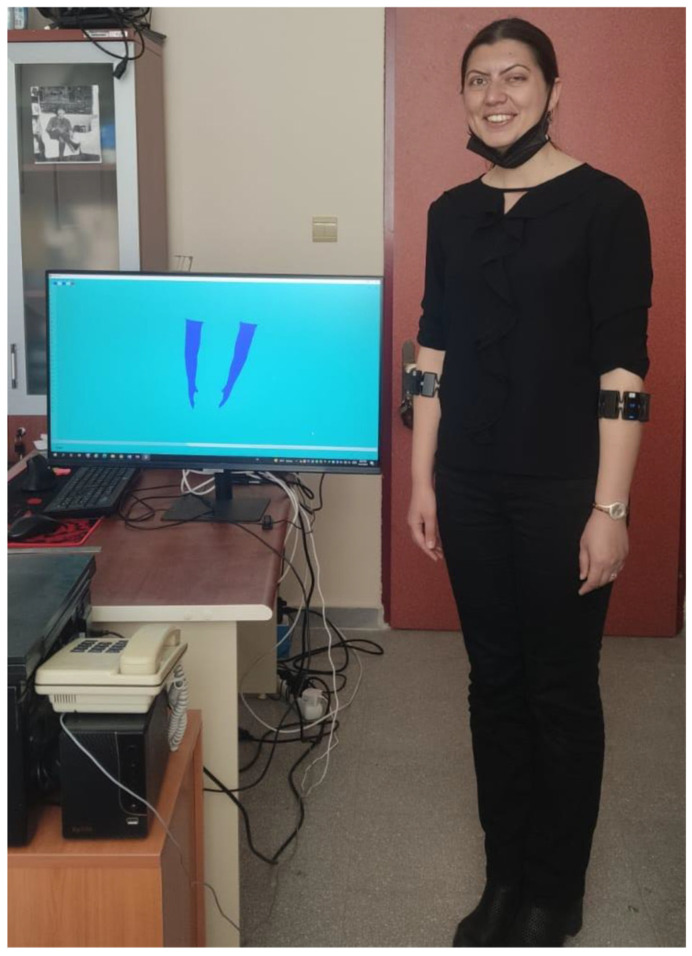
Testing the system with a sign language instructor.

Determining the signs of TSL is a crucial aspect of this study, which the system will test. To achieve this, we received support from Hülya AYKUTLU, a licensed sign language trainer and translator with years of experience in the Special Education Department. The testing location is shown in Figure 5.

The system was designed to predict more than 80 words, but to test its performance, 80 words, which is the maximum number of words used in similar studies, were chosen. Requests from sign language instructors and users were evaluated while selecting these 80 words.

To test the system, 80 frequently used words in sign language and daily speech were selected. The system also supports additional words.

Below are categories for the selected words:

- Greeting and introduction words (hello, I’m glad, meet, etc.), Turkish (merhaba, sevindim, görüşürüz vb.);
- Family words (mother, father, brother, etc.), Turkish (anne, baba, abi vb.);
- Pronouns and person signs (I, you, they etc.), Turkish (ben, sen, onlar vb.);
- Common verbs (come, go, take, give, etc.), Turkish (gel, git, al, ver, vb.);
- Question words (what, why), Turkish (ne, neden);
- Other daily life words (home, name, good, warm, easy, married, year, etc.), Turkish (ev, isim, iyi, sıcak, kolay, evli, yıl vb.).

The 80-word dictionary was repeated 10 times, with each word being recorded by the IMU sensors of the Myo armband device 50 times per second. The data collected include 10 measurements, consisting of gyroscope accelerometer (*x*, *y*, and *z*) and orientation (*x*, *y*, *z*, and *w*). Additionally, data from eight sEMG sensors of the device are measured 200 times per second and stored in memory during recording. At the end of the recording, 1044 features are extracted from the stored data, including raw and feature-extracted data, which are then stored in the database. If the sign recording lasted for 1 s, 4200 raw data and 1044 feature data would be stored. The data were initially segmented using either a fixed time or a fixed reference arm position. Users did not receive the slow and tiring nature of the application testing well. A motion detection system is employed instead of a fixed time to ensure fast and effective communication during data segmentation. The sensitivity setting can be adjusted in the settings section to determine the most suitable option. Another important aspect of this feature is that hearing-impaired individuals may produce signs at varying speeds depending on their level of excitement and emotional state. As a result, the duration of the same sign may differ. Ten different recordings were taken for each received sign to account for this.

### 2.4. Feature Extraction

In machine learning, distinguishable features are crucial, as they enhance the system’s success and performance. The accuracy and performance of the developed SLR system were improved by using feature extraction methods from the EMG data in Table 2 [44]. Some features listed in Table 2 were also utilized for the IMU data.

#### 2.4.1. Time Domain Features

This section describes the application of time domain features, including mean absolute value, root mean square, zero crossing rate, and Willison amplitude. These features provide additional information to that provided by frequency domain features, thereby enhancing the classifier’s performance.

Mean absolute value (*MAV*) is a simple and effective time domain feature that represents the average of the absolute values of the signal.

(2) $$MAV=\frac{1}{N}\sum_{n=1}^{N}\left|X_{n}\right|$$

This equation calculates the average of the absolute values of the signal $X_{n}$, where *N* is the total number of samples. In our study, *MAV* was used to quantify the overall activity level of the sEMG signals. This feature helps distinguish between different muscle activities based on their intensity.

Root mean square (*RMS*) is another commonly used time domain feature that provides a measure of the signal’s magnitude.

(3) $$RMS=\sqrt{\frac{1}{N}\sum_{n=1}^{N}x_{n}^{2}}$$

This equation calculates the square root of the mean of the squares of the signal values $x_{n}$. It provides a measure of the signal’s energy. *RMS* was used to capture the energy content of the sEMG signals, which is important for distinguishing between gestures with different levels of muscle contraction.

Zero crossing rate (*ZCR*) measures the rate at which the signal changes sign, indicating the frequency of signal oscillations.

(4) $$ZCR=\frac{1}{N-1}\sum_{i=1}^{N-1}I[(x_{\left(i\right)}\cdot x_{\left(i+1\right)})<0]$$

Here, I is an indicator function that equals 1 if the product $x_{\left(i\right)}\cdot x_{\left(i+1\right)}$ is less than 0, indicating a zero crossing, and 0 otherwise. *ZCR* was used to measure the frequency content of the sEMG signals in the time domain. This feature is useful for identifying rapid changes in muscle activity.

Willison amplitude (*WAMP*) counts the times the absolute difference between consecutive signal samples exceeds a predefined threshold.

(5) $$WAMP=\sum_{i=1}^{N-1}I[\left(x_{\left(i+1\right)}-x_{\left(i\right)}\right)>\theta ]$$

This equation sums the number of times the absolute difference between consecutive samples $x_{\left(i\right)}$ and $x_{\left(i+1\right)}$ exceeds a threshold θ. *WAMP* was used to count the number of significant changes in muscle activity, providing insights into the frequency and intensity of the gestures. This feature helped distinguish between gestures with different movement patterns.

#### 2.4.2. Frequency Domain and Entropy-Based Features

This section outlines the application of the Fourier transform, wavelet transform, and entropy-based methods for feature extraction. The application of these methods resulted in the generation of a comprehensive and complementary set of features, which collectively enhanced the performance of the classifier.

The Fourier transform is used to convert time domain signals into frequency domain representations. This helps in identifying the dominant frequencies present in the sEMG signals.

(6) $$X_{\left(f\right)}=\sum_{n=1}^{N-1}x_{\left(n\right)}.e^{-j2\pi fn/N}$$

The discrete Fourier transform (DFT) is derived from the continuous Fourier transform but adapted for discrete signals. Here, $x_{\left(n\right)}$ represents the signal in the time domain, and $X_{\left(f\right)}$ represents its frequency domain representation. *N* is the total number of samples, and *j* is the imaginary unit. The Fourier transform was applied to the sEMG signals to convert them from the time domain to the frequency domain. This helped in identifying the dominant frequencies, which are crucial for differentiating between various muscle activities corresponding to different gestures.

The wavelet transform is another method for analyzing signals in both time and frequency domains.

(7) $$W_{\left(t,a\right)}=\int_{-\infty }^{\infty }x_{t^{\prime }}\psi^{*}\left(\frac{t^{\prime }-t}{a}\right)dt^{\prime }$$

The continuous wavelet transform (CWT) involves scaling and translating a wavelet function *ψ*. *t* represents the translation parameter, and *a* represents the scale parameter. $\psi^{*}$ is the complex conjugate of the wavelet function.

The wavelet transform was used to extract time frequency features from the sEMG and IMU signals. These features provided detailed information about the signal’s behavior at different scales and time points, enhancing the classifier’s performance.

Entropy-based features captured the randomness and complexity of the signals. This was important for distinguishing between similar gestures that might have different levels of muscle activity and motion variability.

(8) $$H_{\left(X\right)}=-\sum_{i=1}^{n}P_{\left(x_{i}\right)}logP_{\left(x_{i}\right)}$$

Shannon entropy is derived from information theory. It quantifies the amount of uncertainty or surprise associated with random variables $x_{i}$ and their probabilities $P_{\left(x_{i}\right)}$. Shannon entropy was calculated for each segment of the sEMG and IMU signals to capture the complexity and variability of the muscle activity and motion dynamics. This feature was crucial for distinguishing between different sign language gestures.

The methods used converted all IMU and EMG data from two devices into 1044 pieces of data, resulting in a high level of classification performance. However, training with raw data was not successful.

### 2.5. Classification

In this study, we used various classification methods to address research objectives. The Weka deep learning (WDL) algorithm was employed to harness the power of deep learning for extracting features and classifying data. The K-nearest neighbor (KNN) method, a non-parametric algorithm, was utilized for pattern recognition based on data point proximity in the feature space. We also employed the multilayer perceptron (MLP), a type of artificial neural network, for its ability to model complex relationships within the data through its layered structure. Naïve Bayes (NB), a probabilistic classifier, was chosen for its simplicity and efficiency in managing datasets. The random forest (RF) method, an ensemble learning technique, was applied to combine the predictions from numerous decision trees, improving the classification performance. Support vector machines (SVMs), known for their effectiveness in high-dimensional spaces, were employed to determine the optimal hyperplane to separate data. Each of these classification methods was selected to make use of their specific strengths and capabilities in tackling the complexities of the research problem, enabling a comparative study for a thorough analysis.

This study compared the classification performance of features obtained through feature extraction using various classification algorithms and parameters. All algorithms were tested using the 10-fold cross-validation method. The Weka application programming interface (API), developed specifically for the Windows platform, allows for the use of all algorithms available in Weka by converting the data in the database into ARFF file format [45]. Trainings are saved as a model file containing the algorithm name and parameters. Therefore, a previously performed training can be predicted using the model file without the need for retraining. Only the KNN algorithm is used on the Android platform due to its classification performance and fast operation [46]. The aim is to incorporate additional algorithms in the future, including the Weka API on the Android platform.

To add Weka algorithms to the program, edit the ‘Data\weka\algorithms.txt’ file located in the program’s installation folder. Here is an example of the file’s contents:

bayes.NaiveBayes

lazy.IBk -K 1 -W 0 -A

trees.J48 -C 0.25 -M 2

functions.MultilayerPerceptron -L 0.3 -M 0.2 -N 50 -V 0 -S 0 -E 20 -H 50

trees.RandomForest -P 100 -I 100 -num-slots 1 -K 0 -M 1.0 -V 0.001 -S 1

bayes.BayesNet -D -Q

**Figure 6:**
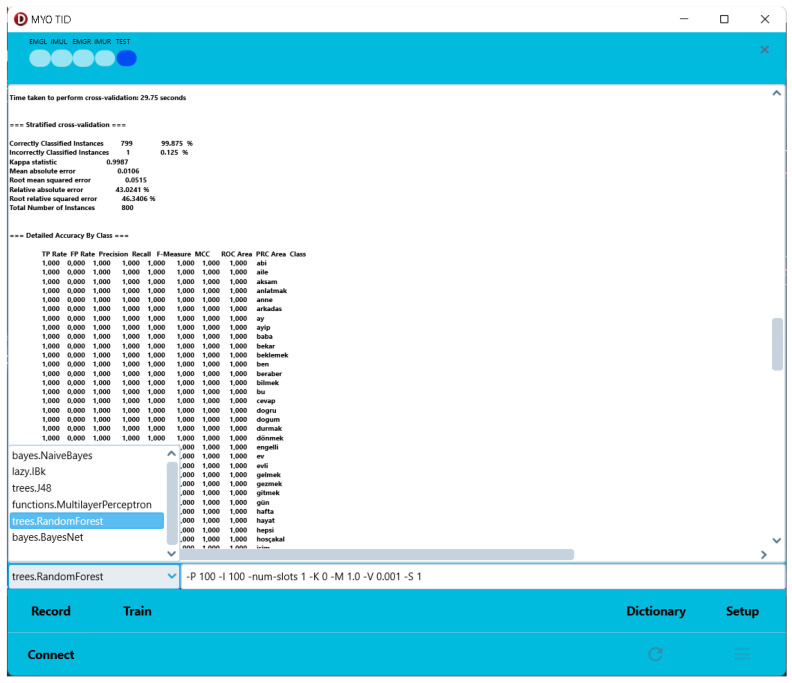
An example Weka training user graphical interface (only Windows).

When the program is executed, each added line is displayed as a combo box, as shown in Figure 6, when the training interface is accessed.

## 3. Results

In the measurements taken for a total of 800 data points, consisting of 80 signs and 10 repetitions, the average time taken for feature extraction from the raw data and classification after the signal ended was 21.2 ms. This demonstrates the system’s ability to perform in real time, as the time was imperceptible to the users testing the system.

The training results of six different algorithms, selected based on their popularity and classification success, were compared. Table 3 presents the results of the training conducted using a total of 800 data points, with 80 signs and 10 records for each sign. The 10-fold cross-validation method was used for testing. This method uses all data for both testing and training. The default parameters of the algorithms in Weka were used for this comparison, as their performance was quite high. The default parameters of the algorithms in Weka were used for this comparison, as their performance was quite high. No alternative parameters were tested.

In another application, the training was conducted by splitting the data at different rates instead of using 10-fold cross-validation. Some of the randomly selected records from 10 records for each sign were used for training, while the remaining records were used for testing. The results of these classifications, made using the same algorithm and parameters, are also shown in Table 4.

The classification results obtained from different algorithms indicate that the random forest algorithm outperformed the naïve Bayes algorithm. It is important to note that training using a single record resulted in very low success rates. Therefore, it is recommended to repeat each sign at least three times to create a record. Increasing the number of repetitions is directly proportional to the increase in performance. Despite the variations in recording speeds, the classification performance remains consistently high.

The variation in performance among different algorithms in a 10-fold cross-validation classification task can be attributed to several factors, such as the algorithms’ nature, their handling of data complexity, and their sensitivity to the specifics of the dataset used. In this section, we will evaluate the performance of the listed algorithms based on three key metrics: accuracy, kappa statistic, and root mean squared error (RMSE).

WDL and RF performance: WDL and RF demonstrated exceptional accuracy of 99.875%, with identical kappa statistics of 0.9987, indicating almost perfect classification capabilities compared to a random classifier. However, it is worth noting that WDL outperforms RF regarding RMSE, with an impressively low value of 0.0053, compared to RF’s RMSE of 0.037. The analysis shows that WDL is more consistent in its predictions across the dataset, possibly due to better handling of outlier data or noise within the dataset.

KNN performs moderately well, with an accuracy of 95.5% and a kappa statistic of 0.9542. It has the lowest RMSE among all algorithms at 0.0020, indicating tight clustering around the true values. KNN is a strong model despite its lower accuracy compared to WDL and RF. It is important to note that KNN may require parameter tuning, such as the choice of ‘k’, and may be sensitive to noisy data.

MLP exhibits a strong performance with an accuracy of 98% and a kappa statistic of 0.9797 despite its relatively higher RMSE of 0.0201. The higher RMSE compared to its accuracy and kappa indicates variations in prediction errors, possibly due to the complexity of the model and the need for careful tuning of its layers and neurons.

NB: In contrast, NB demonstrates the lowest performance among all evaluated models, with an accuracy of 87.625%, a kappa statistic of 0.8747, and a relatively high RMSE of 0.0556. While NB may encounter difficulties when dealing with datasets where features are not independent, which is a core assumption of the algorithm, SVM can handle such datasets.

Although SVM has the highest RMSE of 0.11 among the algorithms, its superior performance in other areas makes it the recommended choice. The analysis demonstrates that the SVM algorithm outperforms the NB algorithm in terms of accuracy and kappa statistics. Although SVM has the highest RMSE of 0.11 among the algorithms, its superior performance in other areas makes it the recommended choice. Although SVM has the highest RMSE of 0.11 among the algorithms, its superior performance in other areas makes it the recommended choice. The high RMSE, despite good accuracy and kappa statistic, suggests that SVM’s decision boundary may be less stable or more sensitive to individual data points, possibly due to the choice of kernel or regularization parameters.

WDL and RF outperform the other models in terms of accuracy and kappa statistics, likely due to their robustness to data imperfections and their ability to model complex patterns. WDL is superior in handling outliers or noise compared to RF, as evidenced by its lower RMSE. The other models’ performance is dependent on their intrinsic assumptions and sensitivity to data characteristics. It is imperative to select the appropriate model based on the specific requirements and nature of the dataset.

## 4. Discussion

In their review article, Kudrinko et al. examined all systematic literature review studies to date and identified the essential features that should be present in SLR systems [47].

- Real-life usability: Sign language technology should be applicable to real-life situations outside of the laboratory.
- Accuracy and minimal delay: The technology must accurately convert sequences of movements into text and speech with minimal delay.
- Aesthetics and comfort: The technology design should prioritize aesthetics and comfort for the user.
- Wireless and rechargeable: To be useful, it must be wireless and easily rechargeable.
- User-centric design: When designing systems.

The existing literature focuses mainly on recognizing sign language movements, but fails to meet these essential criteria, thereby limiting its practical applicability. To address this gap, our study presents a mobile application developed in collaboration with 10 individuals with disabilities who provided valuable feedback confirming its utility and effectiveness in real-life scenarios. In particular, our approach emphasizes personalized application development, recognizing the inherent variability of sEMG data between users. By tailoring the application to individual physiological characteristics, we mitigate the challenges posed by inter-user variability, thereby enhancing performance and usability. In addition, the inclusion of multiple users in the training sets in previous studies may be why classification performance decreases as the number of signs increases, highlighting the importance of personalized approaches to account for individual differences and optimize system performance. Through these efforts, we aim not only to advance the theoretical understanding of SLR but also to provide practical solutions that address the diverse needs of people with hearing impairments, ultimately facilitating seamless communication and integration into society.

The KNN algorithm and its corresponding model were chosen for the mobile application due to their exceptional performance and accuracy. This algorithm has a single effective parameter, the K parameter.

The user can add any number of words to the dictionary. Since biological data such as EMG may vary from person to person, the application is for a single person’s use. When different people’s data have been used for training on the same model in other studies, success rates have been low. The user creates their model by creating their own training data by performing the desired movements. They can adjust the desired delay and sensitivity settings. Since the Text to Speech (TTS) feature in the application uses the TTS system in the operating system, voiceover can be made in the Turkish language. The application allows for users to create their dictionary, store data, and train and use it with the existing artificial intelligence algorithm. In addition, the application allows for users to control the device using sign language, including putting it to sleep, waking it up, and starting and stopping it. All signals are easily recognizable when using the Myo wristbands. The application is now being actively used by disabled Please state the version number of the software.people who participated in the trials.

### Limitations

Despite the promising results of the proposed real-time SLR (v1.0) software, some limitations should be acknowledged.

Firstly, the results presented in Table 3 and Table 4 were obtained from a single subject, limiting the findings’ generalizability. Although the device demonstrated high accuracy and performance for this individual, there is no concrete evidence to suggest that it would perform similarly in a wider population. Future studies will include a more diverse group of subjects to confirm the device’s effectiveness and ensure its applicability to a wider user base. In addition, the between-subject variance will be calculated and reported to provide a full understanding of the variability in the device’s performance.

Secondly, although the software shows potential for use with other sign languages, the current study focuses only on TSL. A shortcoming is the lack of classification results for other languages, such as ASL. Extending the study to more sign languages would provide concrete evidence of the adaptability and reliability of the software in different linguistic contexts.

In conclusion, while the initial results are promising, addressing these limitations in future research will be crucial to validate the effectiveness of the software and increase its robustness and applicability across different user groups and sign languages. In the future, we will redesign the software to use the device we have developed. Although we encountered a challenge during this study with the discontinuation of the Myo armband, we overcame this obstacle by embarking on a project to develop our own device. We aim to create a novel system that comprehensively addresses the needs of people with disabilities. We envision this system not only as a replacement for the Myo armband but also as an innovative solution that surpasses its capabilities. Through this initiative, we aim to democratize access to assistive technology and ensure that people with disabilities around the world can benefit from our system. Our goal is to make this device readily available and accessible to anyone in need, thereby promoting inclusivity and empowerment within the disability community.

## Figures and Tables

**Table 1:** Comparison of existing it based solutions.

Device/Technology	Accuracy (Around)	Mobility	User Convenience
Kinect	90%	Not a mobile solution	User must stand in front of the sensor
Data glove	80%	Not a mobile solution	User must wear device
Leap Motion	95–98%	Not a mobile solution	User must stand in front of the sensor
Image processing	90%	Not a mobile solution	User must stand in front of the camera

**Table 2:** Features extracted from sEMG signals.

Time Domain	Frequency Domain	Entropy-Based
Mean absolute value	Peak frequency	Shannon
Integrated EMG	Median frequency	Spectral
Higuchi fractal dimension	Modified median frequency	SVD
Petrosian fractal dimension	Modified mean frequency	Fisher
Detrended fluctuation analysis	Intensity weighted mean frequency	
Nonlinear energy	Intensity weighted bandwidth	
Slope	Total spectrum	
Line length	Mean power spectrum	
Willison amplitude	Wavelet energy	
Standard deviation	AR coefficient 1	
Min value	AR modelling error 1	
Max value	AR coefficient 2	
Hurst exponent	AR modelling error 2	
Minima	AR coefficient 4	
Maxima	AR modelling error4	
Skewness	AR coefficient 8	
Kurtosis	AR modelling error 8	
Zero crossings		
Zero crossings of 1 derivative		
Zero crossings of 2 derivatives		
RMS amplitude		
Inactive samples		
Mobility		
Activity		
Complexity		

**Table 3:** Tenfold cross-validation classification performances of different algorithms.

Algorithm	Accuracy (%)	Kappa Statistic	Root Mean Squared Error
Weka deep learning (WDL)	99.8750	0.9987	0.0053
K-nearest neighbor (KNN)	95.5000	0.9542	0.0020
Multilayer perceptron (MLP)	98.0000	0.9797	0.0201
Naïve Bayes (NB)	87.6250	0.8747	0.0556
Random forest (RF)	99.8750	0.9987	0.037
Support vector machine (SVM)	97.6250	0.9759	0.11

**Table 4:** Classification performances of different algorithms with split data.

Number of Records Used		Algorithm Accuracy (%)					
Training	Test	MLP	WDL	RF	KNN	SVN	NB
1	1	73.75	62.5	30	71.25	62.5	16.25
2	2	90.625	73.75	89.375	78.125	81.25	26.875
3	3	91.25	99.5833	99.1667	89.166	90	45.833
4	4	92.1875	99.375	98.75	91.25	90	61.875
5	5	95	99.75	99.25	92.25	94	67.75
6	4	95	91.5625	99.0625	95	95.312	75.312
9	1	97.5	96.25	100	97.5	98.75	85

## Data Availability

Access to these data are restricted, but interested researchers may contact Assoc. Prof. İlhan UMUT (iumut@nku.edu.tr) for inquiries regarding data access.
